# Evaluation of Calibration Equations by Using Regression Analysis: An Example of Chemical Analysis

**DOI:** 10.3390/s22020447

**Published:** 2022-01-07

**Authors:** Hsuan-Yu Chen, Chiachung Chen

**Affiliations:** 1 Africa Industrial Research Center, National Chung Hsing University, Taichung 40227, Taiwan; wakaharu37@gmail.com; 2 Department of Bio-Industrial Mechatronics Engineering, National Chung Hsing University, Taichung 40227, Taiwan

**Keywords:** calibration equation, regression analysis, nonlinearity, prediction, outliers

## Abstract

A calibration curve is used to express the relationship between the response of the measuring technique and the standard concentration of the target analyst. The calibration equation verifies the response of a chemical instrument to the known properties of materials and is established using regression analysis. An adequate calibration equation ensures the performance of these instruments. Most studies use linear and polynomial equations. This study uses data sets from previous studies. Four types of calibration equations are proposed: linear, higher-order polynomial, exponential rise to maximum and power equations. A constant variance test was performed to assess the suitability of calibration equations for this dataset. Suspected outliers in the data sets are verified. The standard error of the estimate errors, *s*, was used as criteria to determine the fitting performance. The Prediction Sum of Squares (*PRESS*) statistic is used to compare the prediction ability. Residual plots are used as quantitative criteria. Suspected outliers in the data sets are checked. The results of this study show that linear and higher order polynomial equations do not allow accurate calibration equations for many data sets. Nonlinear equations are suited to most of the data sets. Different forms of calibration equations are proposed. The logarithmic transformation of the response is used to stabilize non-constant variance in the response data. When outliers are removed, this calibration equation’s fit and prediction ability is significantly increased. The adequate calibration equations with the data sets obtained with the same equipment and laboratory indicated that the adequate calibration equations differed. No universe calibration equation could be found for these data sets. The method for this study can be used for other chemical instruments to establish an adequate calibration equation and ensure the best performance.

## 1. Introduction

The performance characteristics include accuracy, precision and sensibility of the sensors or instrument is so important, especially in chemical analysis [1,2,3]. Most quantitative analytical techniques for chemical analysis, such as spectrometry, Inductively Coupled Plasma Mass Spectrometry (ICP-MP) or electrophoresis, require a calibration curve to express the relationship between the response of the measuring technique and the standard concentration of the target analyst [4,5].

According to the definition of Dux, the calibration equation is used to verify the response of an instrument to the known properties of a material [6]. In terms of the user of the instrument, the structure of the instrumentation is so complex that it is difficult to adjust. Calibration detects the response using a series of samples of the known concentration and sufficient purity. A calibration curve is established to express the relationship between the response and the standard concentration for physical, chemical, and biological sensors [7,8,9]. The calibration curve is fitted using regression analysis to fit different models to experimental data [10,11].

A calibration equation expresses the quantitative relationship between the response of an analytical technique and the standard concentrations of the target. The responses of instruments include current, potential, peak height, peak area or peak ratio. The best equation to represent the calibration curve is selected using regression analysis. The response of the analytic instrumentation is the dependent variable (*y*) and the standard concentration is the independent variable (*x*). The equations used for analysis are linear equations, polynomial equations, weighted linear equations, and linear models with a logarithmic transformation of variables [10,11,12,13].

Several criteria are used to evaluate fitting-agreement for a calibration equation, such as the determination of coefficient (*R*^2^), the adjusted determination coefficient (*R_adj_*^2^), the Akaike’s Information Criteria (*AIC*) and an Analysis of Variance (*ANOVA*). Residual plots are used as a qualitative criterion to determine the suitability of a calibration equation [10,11,12,13,14].

Rodriguez et al. used a linear regression model to establish a calibration equation for spectrophotometric, spectrofluorometric and chromatographic methods and the *R*^2^ and the residual standard deviation of regression were the criteria [14]. Huber proposed three equations: linear equations and polynomial equations without intercept and logarithmic *y* and *x* values to measure wide ranges and low concentrations [15]. Mulholland and Hibbent studied the calibration equation for High-Performance Liquid Chromatography (HPLC) and found that a heteroscedastic data distribution with a linear equation results in significant unexpected errors [16]. The study determined that a reliable method to validate an inadequate model is necessary to reduce errors. Desimoni used a weighted linear regression to address heteroscedastic issues in response data [17]. The weights for the gression analysis are calculated using the standard deviation of the linear function and the outliers are checked using an *F*-test. However, only three replicates were measured for each standard concentration.

Linear equations, *y* = *a* + *bx*, quadratic equations, *y* = *a* + *bx* + *cx*^2^ and a non-linear equation, *y* = *a* + *bx^c^* were used to evaluate calibration equations for chromatography and spectroscopy by Kirkup and Mulholland [18]. The study determined that quadratic and nonlinear equations produce a better fitting agreement than linear equations because the calibration curves are slightly curved. Bruggemann et al. tested the nonlinear calibration equations using a lack-of-fit test and showed that a polynomial calibration equation gives good results [19].

*R*^2^, the standard errors of the estimate values *s* and visual inspection of residual plots are standard criteria. Lavagnini and Magno used a statistical technique to establish univariate calibration for gas chromatography/mass spectrometry (GC-MS) and used a higher-order polynomial equation for nonlinear curves [20]. The residual plots are used to assess the heteroscedastic data. Ortiz et al. used univariate regression for calibration curves and proposed a quadratic polynomial equation for nonlinear calibration curves. The regression results were verified using an *ANOVA*, a lack of fit test, and residual plots [21].

Rozet et al. used *R*^2^, *R_adj_*^2^, and AIC, small sample adjusted information criteria (AICc) and Bayesian Information Criteria (BIC) to select calibration equations [8]. Rawski et al. used a statistical method to evaluate linear and quadratic equations for calibration curves [22]. The validation uses *R*^2^, a lack of fit test and the *F*-test. Desharnais et al. proposed selecting and validating a calibration equation [23]. The variance in a linear equation is used to evaluate the constant variance and calculate the weights for a weighted regression equation. A partial *F*-test is used to select the order of the polynomial equation. Martin et al. used residual analysis to verify the fitting-agreement for calibration equations and a constant variance [24]. Several studies involve nonlinear calibration curves but polynomial equations are only used to represent the data distribution [25].

Calibration equations are still the subject of many studies. Higher-order polynomial equations have been used to determine uncertainty and the limit of detection in Label-free biosensors [26]. Machado et al. used a linear calibration equation for light elements in animal tissues and plants and the criteria for model evaluation were *R*^2^ and *s* [27]. Pagliano and Meija established a calibration equation for isotope dilution mass spectrometry (IDMS) and noted that the *R*^2^ values exceed 0.99 for all cases and the calibration curves show a type of linear relationship [28].

For a sensitivity analysis of two types of surface plasmon resonance (SPR), Mrozek et al. used a linear equation to express the relationship between SPR signals and the Cathepsin S (CatS) concentration [29].

If the calibration curves have a relatively narrow range, a linear equation can be used as a calibration equation [25]. Hinshaw constructed several calibration curves for gas chromatography (GC) and showed that higher-order polynomial equations give a better fit than a linear equation, especially for lower concentrations [26]. Frisbie et al. used a quadratic polynomial regression equation instead of a linear equation for analytical chemistry [30].

Martin et al. studied the calibration equations for several compounds detected using Liquid chromatography-tandem mass spectrometry (LC-MS/MS) and used a linear equation for smaller concentrations [31]. However, there is no universal model for all cases. To ensure reliable Selective Reaction Monitoring/Multiple reaction monitoring-mass spectrometry (SRM/MRM-MS)-based proteomic assays, Kohl et al. proposed a complex calibration equation [32], *y’* = *c*_0_ + *c*_1_*Exp*(*c*_2_*x’*), wherein *y’* and *x’* are the logarithmical response area (*y*) and logarithmical concentration (*x*).

The *R*^2^ value is used as a criterion to evaluate calibration equations, but this criterion is not supported by theory and the numerical value can be manipulated easily [33,34,35,36,37]. There is also no acceptable value for *R*^2^. Mulholland and Hibbert noted that some studies use an *R*^2^ value between 0.99 and 1.0 as an acceptable criterion, which is inadequate for chemometric fields [16]. In terms of the effect of the number of parameters, the *R_ad_j*^2^ criterion was proposed by Kirkup and Mulholland [18], and Rozet et al. [5]. However, this criterion proved flawed [8], so the *R*^2^ value cannot be the only criterion. The criterion did not be used in this study.

The sum of the square residuals (SSR) and *s* are used to evaluate the fit of calibration models. The *s* value has the same unit as the response for detecting techniques, so it is a useful criterion [33,35,36].

The lack of fit technique is used to test the validation of linear equations, but this technique gives no information about the order of a polynomial equation [33,38,39,40]. An *ANOVA* is used to test the significance of the effect of *x* (standard concentration) on the *y* (response of detecting technique) but does not determine the adequacy of a calibration equation.

The detection of outliers in a calibration equation is important. The existence of outliers affects the fit of the calibration equation and the estimated values of parameters in the calibration equation. Njaka et al. detected outliers in linear calibration equations in a study of graphite furnace atomic absorption spectrometry (GF-AAS) and concluded that movement outliers increases the quality of the measurement [41].

A calibration procedure involves several measurements at specific concentrations. As concentration increases, the distribution of measurement data at this concentration is increased, so the variance for each concentration is not constant. This constitutes heteroscedastic data. A weighted regression is used to address this problem [17,19,42,43]. The value of the weight must be determined for a weighted regression. This value is calculated using the reciprocal standard deviation of the error for the measurement data at each concentration. The practical difficulties of this calculation are that replicates are required at each level [36], and there must be more than nine samples to ensure validity [33,44]. However, this requirement is not a feature of previous studies. If the weight is estimated incorrectly, the result of the weighted regression is less accurate than the result using an unweighted regression [33,39].

In terms of regression techniques, the prediction ability of calibration equations is important. Criteria have been proposed to evaluate the prediction performance but the predictive ability of calibration equations is not a feature of studies that assess calibration equations for chemical analysis [33,40].

A residual plot is used to validate a regression analysis. A visual method is ambiguous if the number of data is limited. Another quantitative criterion must be considered. A single session coefficient is tested to determine the order of a polynomial equation. Outliers in the data needs are evaluated, and the effect of outliers on the regression analysis requires further study [33,34,39,40].

To the authors’ best knowledge, regression techniques have not been fully used to study calibration equations for chemical analysis. This study determines calibration equations for chemical analysis using regression analysis. The data is collected from previous studies.

## 2. Materials and Methods

### 2.1. Regression Analysis

For this study, the dependent variable *y_i_* is the response of the instrument of chemical analysis. The independent variable *x_i_* is the standard concentrations of the target measurement.

The calibration methods for this study are:

1. Linear equations
  *y* = *a*_0_ + *a*_1_*x* (1)
2. Higher order polynomial equations
  *y* = *b*_o_ + *b*_1_*x* + *b*_2_*x*^2^ + … + *b*_*k*_*x*^*k*^ (2)
3. Exponential rise to maximum equations (ERTM equations)
  *y* = *c*_1_ (1 − *Exp*(*c*_2_*x*)) (3)
4. Exponential rice to maximum equations with intercept
  *y* = *d*_o_ + *d*_1_ (1 − *Exp*(*d*_2_*x*)) (4)
5. Power equations
  *y* = *e*_1_*x*^*e*2^ (5)
6. Power equations with intercept
  *y* = *f*_o_ + *f*_*i*_*x*^*f*2^ (6)

If the *y_i_* data is heteroscedastic, the dependent variable is transformed to stabilize the variance. This study uses a logarithmic transformation. The variable *y* for Equations (1)–(6) is replaced by *lny.* These new equations are:

*lny* = *a*_0_ + *a*_1_*x* (7)

*lny* = *b_o_* + *b*_1_*x* + *b*_2_*x*^2^ + … + *b*_*k*_*x*^*k*^ (8)

*lny* = *c*_1_ (1 − *Exp*(−*c*_2_*x*)) (9)

*lny* = *d_o_* + *d*_1_ (1 − *Exp*(−*d*_2_*x*)) (10)

*lny* = *e*_1_*x*^*e*2^ (11)

*lny* = *f_o_* + *f*_*i*_*x*^*f*2^ (12)

Statistical analysis uses Sigma plot V.14.0 (SPSS Inc., Chicago, IL, USA).

### 2.2. Evaluation Criteria for Calibration Equations

#### 2.2.1. The Criteria of Fitting-Agreement

The standard error in the estimate errors, *s* is use as criteria to assess the fit:

(13) $$s=\;\frac{\sqrt{{\left(y_{i}-{\hat{y}}_{i}\right)}^{2}}}{n-p}$$

where *y_i_* is the dependent variable, ${\hat{y}}_{i}$ is the predicted value for the calibration equation, *n* is the number of data points and p is the number of parameters.

#### 2.2.2. Criteria for Prediction

The Prediction Sum of Squares (*PRESS*) statistic is used to compare the prediction ability for different equations [33,35,39,45]. If the data for responses and standard concentrations consist of *n* observations, the first observation (*x*_1_, *y*_1_) is removed from the data set. The remaining *n* − 1 observations are used to estimate the values of parameters for a specific equation. The value for the first observation (*x*_1_) is then substituted into this first specific equation to calculate the predicted value. This predicted value is denoted as ${\hat{y}}_{1,-1}$, The predictive error for (*x*_1_, *y*_1_) is calculated as *y*_1_- ${\hat{y}}_{1,-1}$ and denoted as *e*_1,−1_.

The first observation (*x*_1_, *y*_1_) is then replaced in the data set, and the second observation (*x*_2_, *y*_2_) is withdrawn. The new parameters for this specific equation are then estimated again. *x*_2_ is substituted into the second specific equation to calculate the predicted value, ${\hat{y}}_{2,-2}$. The predicted error in the second observation is calculated as *y*_2_ − ${\hat{y}}_{2,-2}$ or *e*_2,−2_. Using this method, each observation is removed and the predicted error is calculated. The n prediction error is called the PRESS residuals, and is denoted as $y_{i}-{\hat{y}}_{i,-i}={\hat{e}}_{i,-i}$. Observation (*x_i_*, *y_i_*) is not used to determine the fit and evaluate the predictive ability simultaneously, so the evaluations for fit and prediction are independent. The statistic is defined as *PRESS*:

(14) $$\mathrm{PRESS}=\sum {\left(y_{i}-{\hat{y}}_{i,-i}\right)}^{2}=\sum ({\hat{e}}_{i,-i}{}^{2})$$

For different calibration equations, the smaller the value of *PRESS*, the better is the prediction ability.

### 2.3. Residual Plots

Residual plots are the plots of residuals versus the predicted values. If the errors have a uniform distribution along the *y_i_* = 0 line, the regression model is adequate. If the variance of the errors increases as the prediction increases, such as a funnel distribution, the error variance is not constant (heterogeneous variance). If the error distribution has a fixed pattern, the equation is not adequate. This occurs if a nonlinear curve is treated using linear regression.

### 2.4. Constant Variance Test

If the number of data points is limited, visual observation cannot be used to determine the variance of errors. The Spearman Rank correlation between the observed values of *y_i_* and the absolute residual values is calculated. This statistic is then used to determine the relationship between the two variables.

### 2.5. Transformation

If the variance is not constant, the dependent data is transformed to stabilize the error variance. This study uses a logarithmic transformation (*lny*). The independent data is zero for a blank concentration. This cannot be transformed to a logarithmic form (*lnx*) or an inverse power form (1/*x*), so independent data (*x_i_*) is not transformed for this study.

### 2.6. The Test on a Single Regression Coefficient

To determine whether a variable is significant, the *t*-value for the parameter for the calibration equation is tested.

The hypothesis is:

(15) $$H_{0}-b_{i}=0$$

(16) $$H_{1}-b_{i}\neq 0$$

The *t*-value of *b_i_* is calculated as:

(17) $$t=b_{i}/se(b_{i})$$

where *b_i_* is the parameter value and *se*(*b_i_*) is the standard error.

### 2.7. Outlier Test

The criteria for outliers is that the standardized residual of *y_i_* has a critical value > 2.5 and the difference in fit in standard (*DFFITS*) has flag values > 2.0 [33,38].

### 2.8. Data Sources for Calibration Curves

Seventeen data sets are used to determine the adequacy of calibration equations, and the results are shown in Table 1. All original data for the response of chemical analysis instrumentations and standard concentrations from previous studies.

## 3. Results

After evaluating the adequacy of equations for that are listed in Table 1, the results of the regression analysis involve four types of calibration equations:

a. Linear equations
b. Nonlinear equations
c. Calibration equations with non-constant variance
d. Calibration curves with outliers

### 3.1. Linear Equations

**Figure 1:**
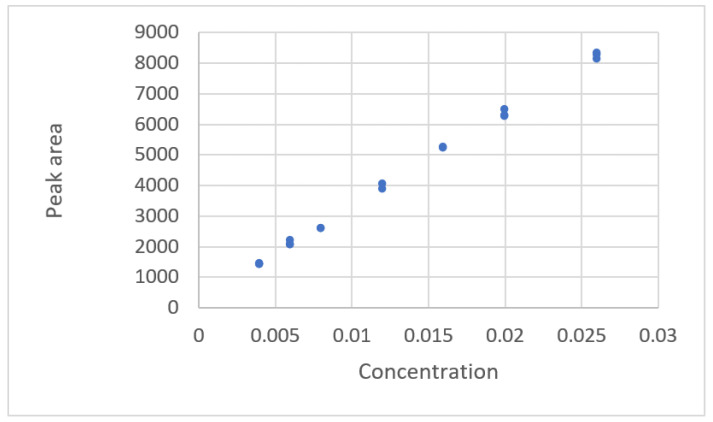
The relationship between the ascorbic concentration and the peak area for HPLC (data published [21], EX.1).

**Figure 2:**
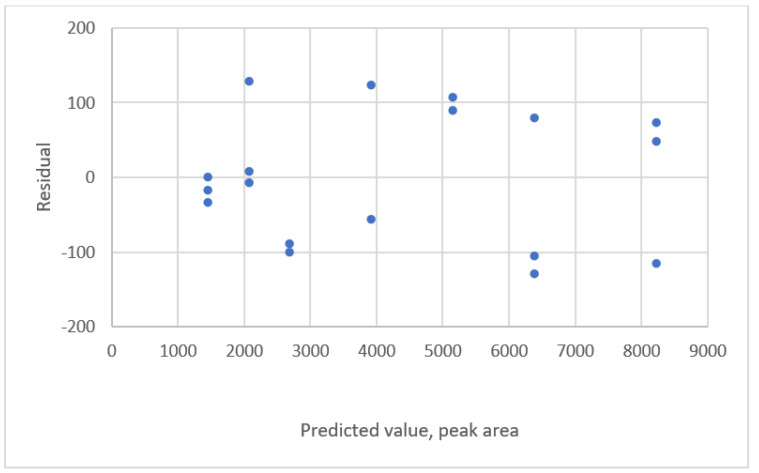
Residual plot for concentration and peak area using a linear calibration equation for the data of Ortiz et al. ([21], Ex.1).

The type of data distribution for a linear equation ([21], Ex.1) is shown in Figure 1. This shows the relationship between the ascorbic concentration and the peak area of HPLC. The results of the evaluation of fit for calibration equations and the criteria are listed in Table 2. The results show that all equations are adequate. The residual plots for these equations feature a uniform distribution. The residual plot for the linear calibration equation is shown in Figure 2.

The linear equation has the smallest *s* value, so the fitting agreement is best. The *PRESS* value for this linear equation is less than the value for other equations, so it gives the best prediction. For the data distribution between cadmium concentration and the current response using Anodic stripping voltammetry ([21], Ex.2), two calibration equations are adequate:

*y* = −0.416 + 0.263*x*, *s* = 0.279, *PRESS* = 2.392 (18)

The other four calibration equations (Equations (3)–(6)) give residual plots with fixed patterns. The *t*-test for the numerical value of 0.000603 for Equation (18) is valid.

*y* = 0.436 + 0.214*x* + 0.000603*x*^2^, *s* = 0.287, *PRESS* = 2.291 (19)

The other four calibration equations (Equations (3)–(6)) give residual plots with fixed patterns. The *t*-test for the numerical value of 0.000603 for Equation (19) is valid.

The quadratic polynomial equation has a smaller *PRESS* than the linear equation, giving a better prediction. The linear equation gives a better fit because the *s* value is smaller. Both are adequate calibration equations. A previous study ([21], Ex.2) used only a linear equation.

### 3.2. Nonlinear Equations

#### 3.2.1. Quadratic Equations

**Figure 3:**
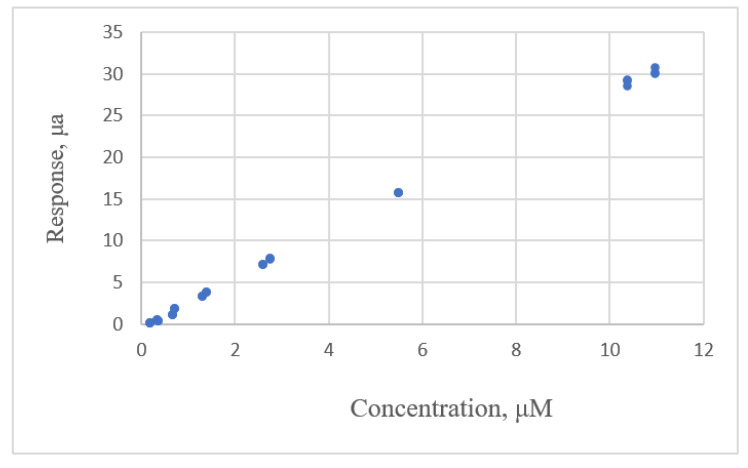
The relationship between sulfide concentrations and the response for flow injection analysis (data published [17]).

**Figure 4:**
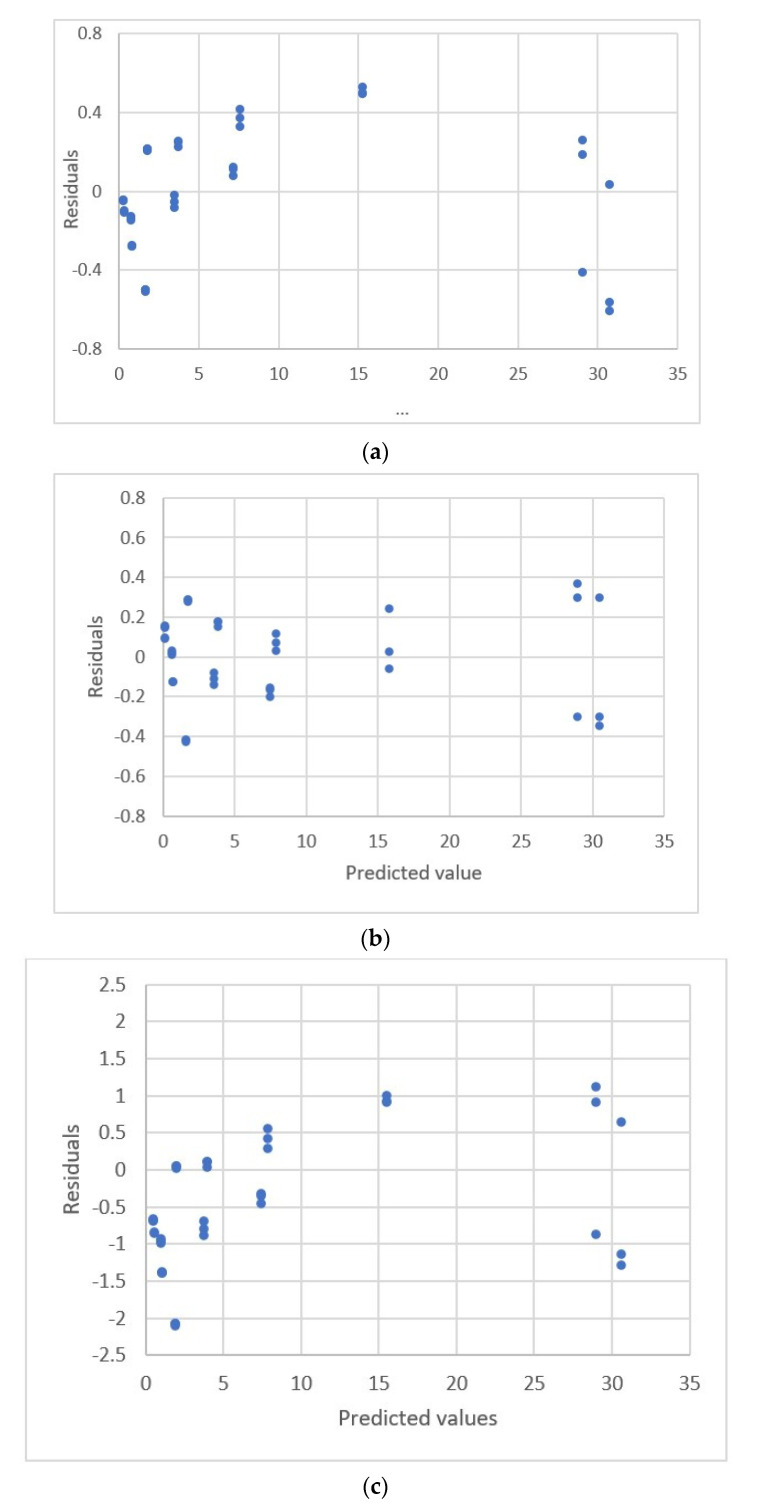

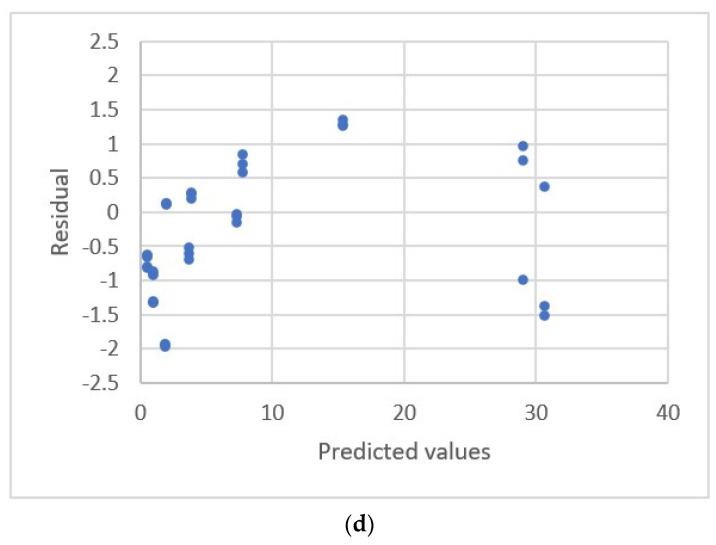
Residual plots for concentration and peak area using calibration equations for the data [17]. (**a**) Linear equation. (**b**) Second polynomial equation. (**c**) Exponential rise to the maximum equation. (**d**) Power equation.

The relationship between sulfide concentrations and the response for flow injection analysis is shown in Figure 3. The results for the estimated parameters and comparative statistics for six equations are shown in Table 3. The residual plots for these equations are shown in Figure 4. The linear equation, the ERTM equation, and the power equation exhibit a fixed pattern for the residual distribution, so these three calibration equations are not adequate. The quadratic polynomial equation, the ERTM equation with intercept, and the power equation with intercept exhibit a uniform distribution for residuals.

The quadratic polynomial gives the best fit (the smallest of *s* value) and the best prediction performance, with the smallest value of *PRESS*. This data set is from a study by Desimoni [17]. This study uses a linear equation as the sole model and assessment use the sole criterion of the *R*^2^ value. In this study, we use more models for comparison and show that the model gives a better fit and prediction than the linear equation that is used in the previous study [17].

#### 3.2.2. The 4th Order Polynomial Equations

**Figure 5:**
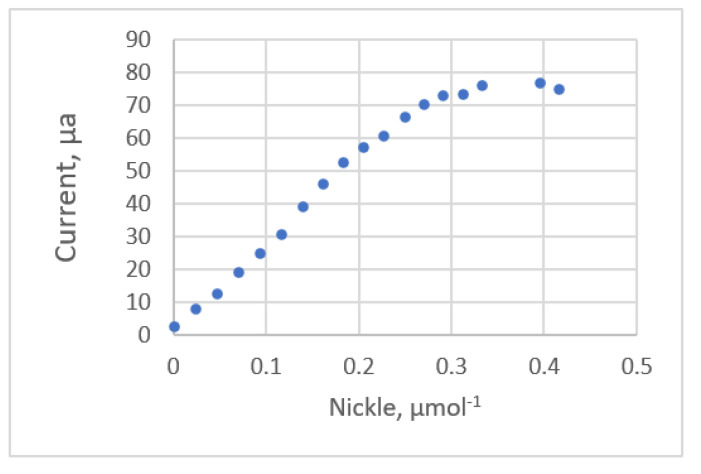
The relationship between nickel concentration and the current for square-wave adsorptive-stripping voltammetry (data published [21] Ex.3).

**Figure 6:**
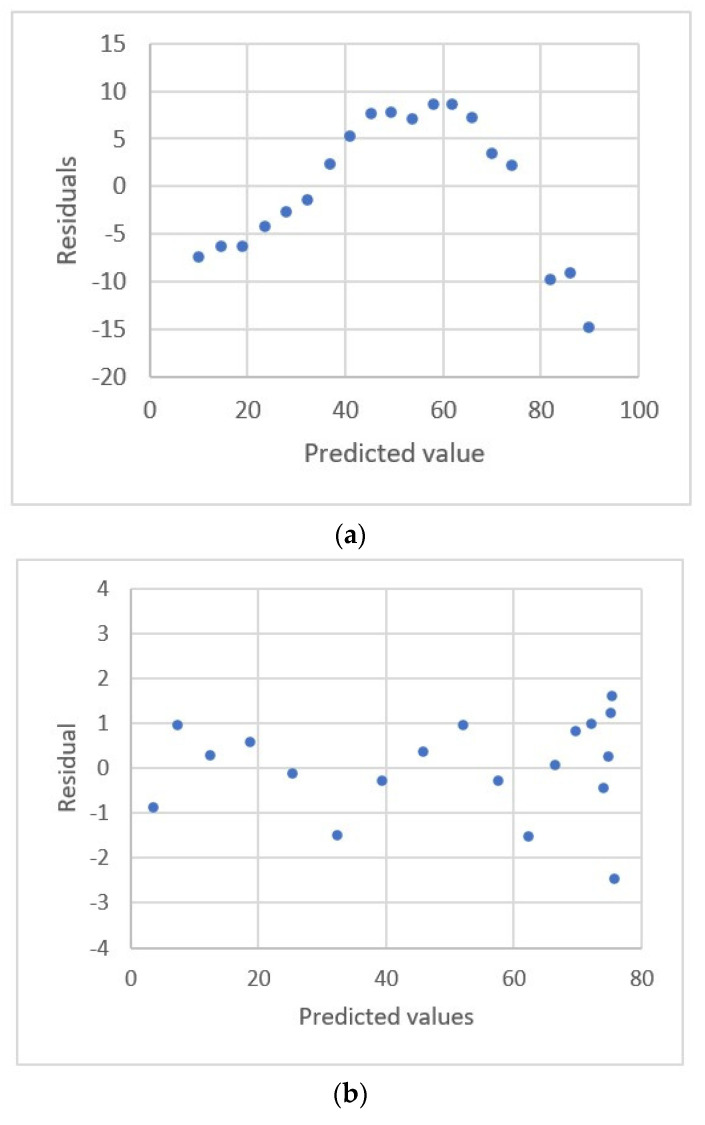
Residual plot for nickel concentration and peak area using calibration equations for the data [21] (Ex.3). (**a**) Linear equation. (**b**) 4th order polynomial equation.

The relationship between the nickel concentration and the current for square-wave adsorptive-stripping voltammetry ([21], Ex.3) is shown in Figure 5. Table 4 lists the estimated parameters and the comparative statistics for these calibration equations. The 4th order polynomial equation gives the lowest value for *s* and *PRESS*. Only this equation gives a uniform distribution for the residual plots. The residual plots for the other seven equations exhibit a systematic pattern. The residual plots for the linear and fourth-order polynomial equations are shown in Figure 6. The respective values for the fitting criterion, *s*, for the quadratic, the third-order and the fourth-order polynomial equations are 2.986, 1.707 and 1.360. The respective values for the prediction criterion, *PRESS*, for the quadratic, the third-order and the fourth-order polynomial equations are 231.49, 81.364 and 70.125. The results show that an adequate calibration equation gives a significantly better fit and prediction.

The study by Oritz et al. ([21], Ex.3) analyzed this data set using the least square (LS) and least median squares method (LMS) in the form of a linear equation. The result of this study shows that the third-order polynomial equation gives a better fit than the two equations that were proposed by Oritz et al. [21]. As the concentration levels increase, significant deviation errors develop for the LMS calibration equation, so the fit and prediction are poor.

#### 3.2.3. Exponential Rise to Maximum Equations

**Figure 7:**
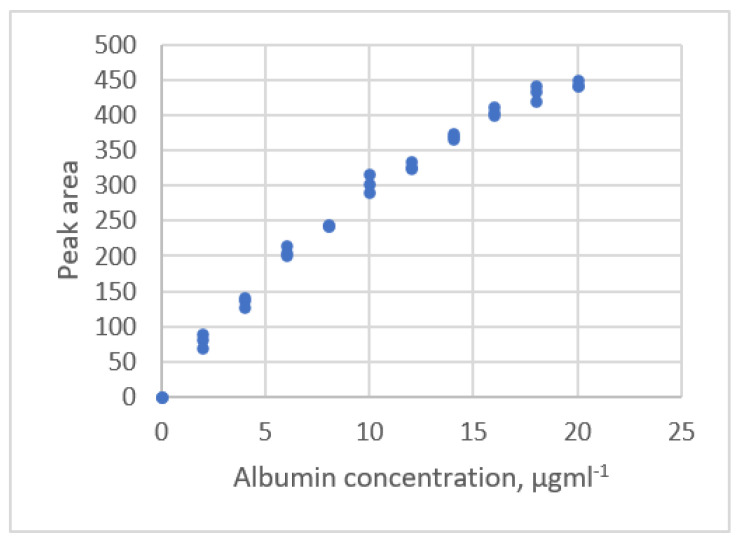
The relationship between albumin concentration and the response for spectrophotometric measurement (data published [22]).

**Figure 8:**
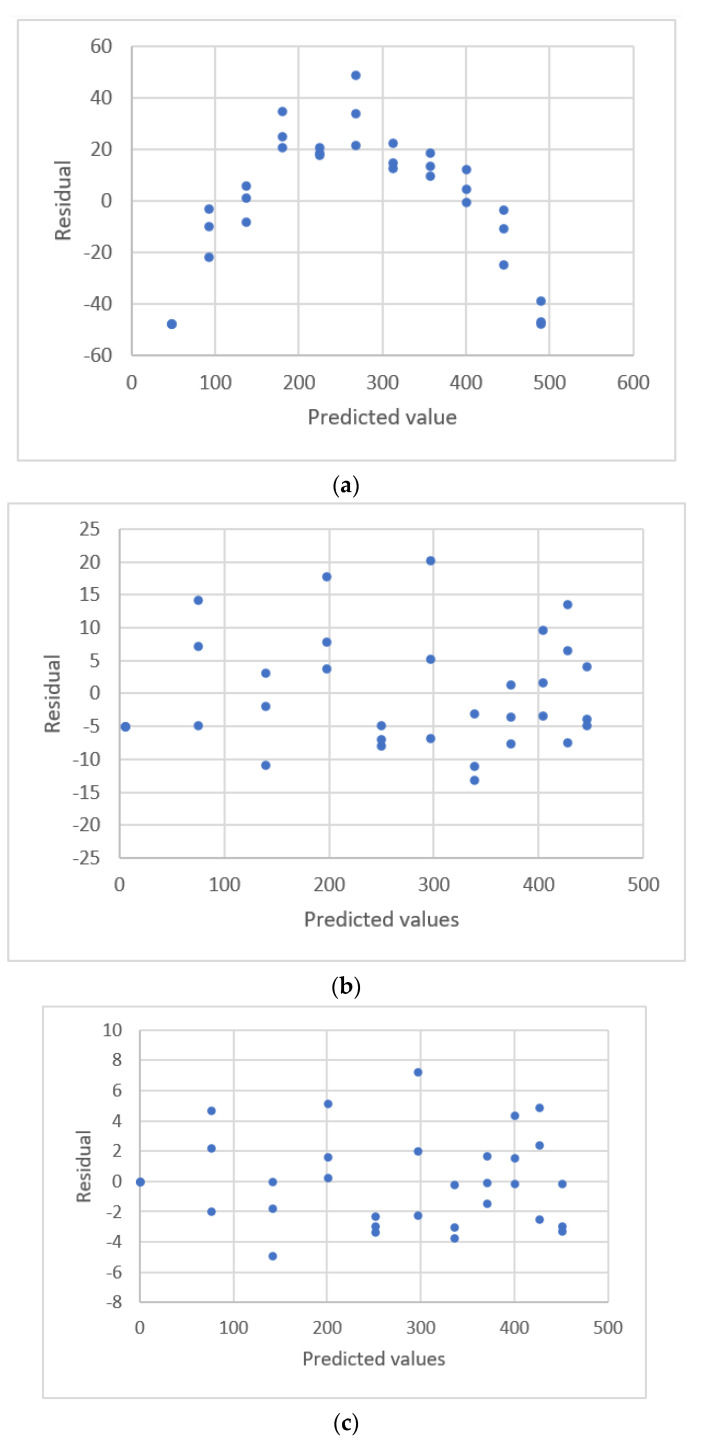

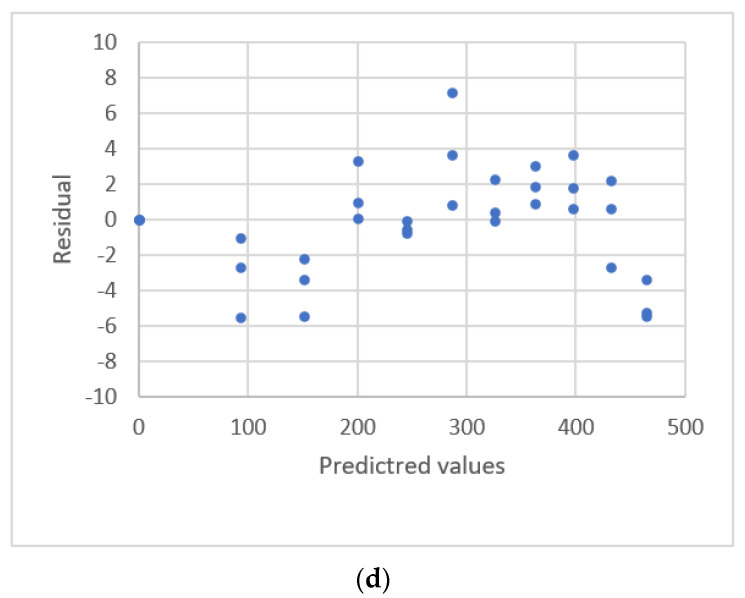
Residual plot for albumin concentration and the response for spectrophotometric measurement using linear calibration equations for the data [22]. (**a**) Linear equation. (**b**) Polynomial equation. (**c**) Exponential rise to maximum equation. (**d**) Power equation.

The data distribution for Albumin concentration and the response for the spectrophotometric measurement [22] are shown in Figure 7. The estimated parameters and the comparative criteria are listed in Table 5. The quadratic polynomial equation, the ERTM equation and the ERTM equation with intercept are adequate. The residual plots for these equations are shown in Figure 8. The respective values for the fitting criterion, *s*, for the quadratic polynomial equation, the ERTM equation and the ERTM equation with intercept are 8.766, 8.560 and 8.698. The respective values for the prediction criterion, *PRESS*, for these three equations are 2718, 2611 and 2672. Compared with the linear equation, the results show that the ERTM equation gives a significantly better fit and prediction.

The ERTM equation has the lowest value for *s* and *PRESS*. This equation gives the best fit and prediction of all equations.

The study by Rawski et al. [22] used the lack-of-fit test for this data set to evaluate the linear and quadratic polynomial equations and showed that the second-order polynomial equation gives a better fit than the linear equation. This study uses other forms of equations and shows that the second polynomial equation is adequate, but the ERTM equation gives a better fit and prediction.

#### 3.2.4. Power Equations

**Figure 9:**
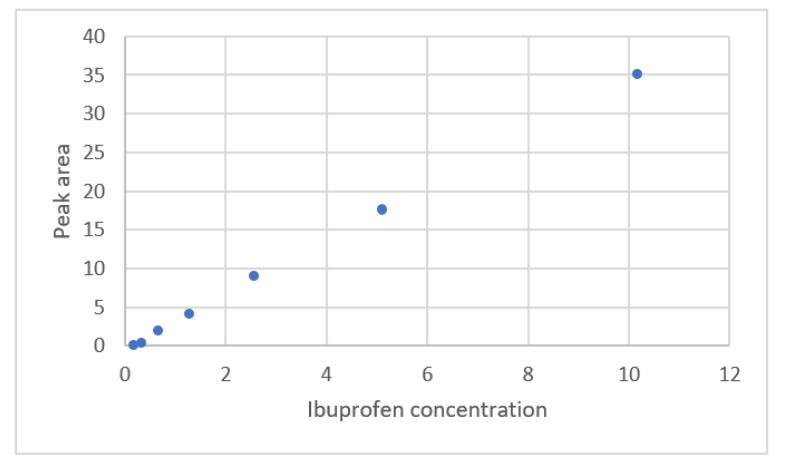
The relationship between ibuprofen concentration and the peak area for HPLC (data published [18]).

**Figure 10:**
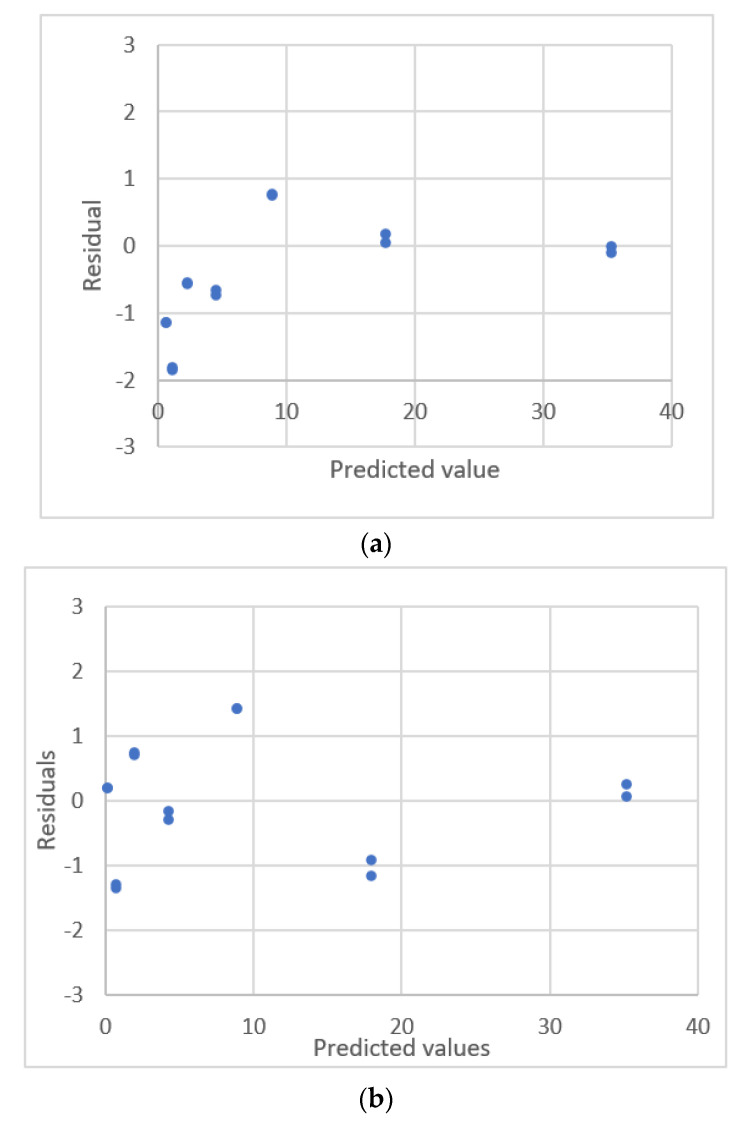

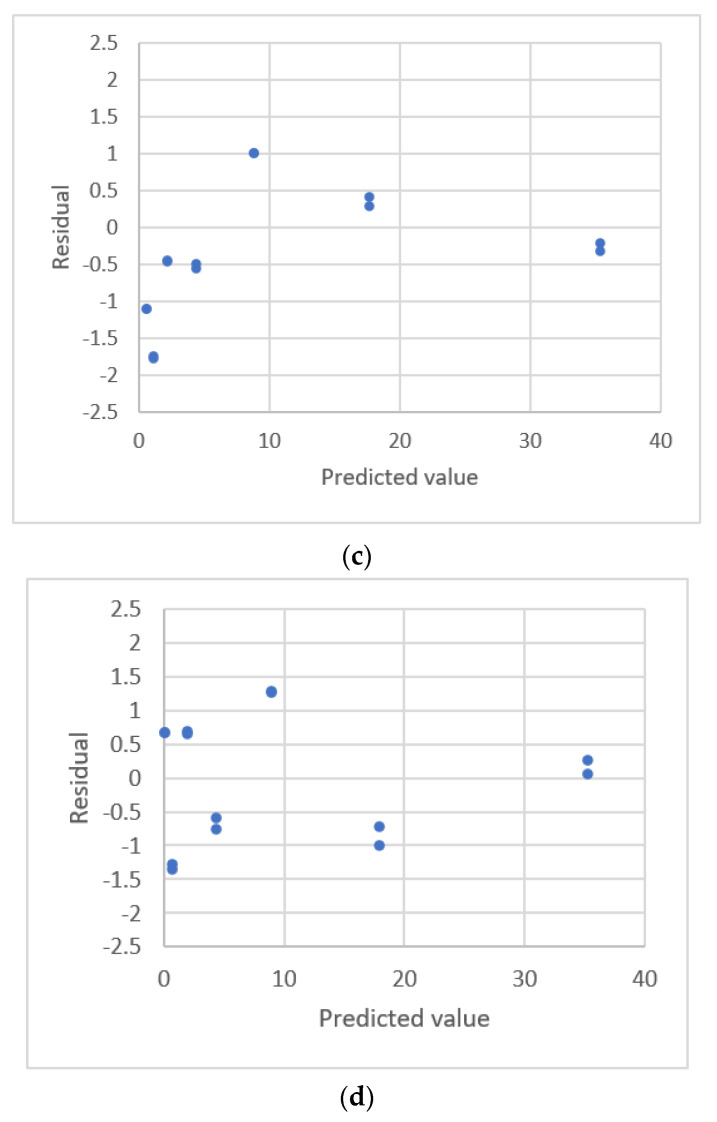
Residual plot for ibuprofen concentration and the peak area for HPLC using calibration equations for the data [18]. (**a**) Exponential rise to maximum equation. (**b**) Exponential rise to maximum equation with intercept. (**c**) Power equation. (**d**) Power equation with intercept.

The calibration curve for ibuprofen concentration and the peak area for HPLC was plotted by Kirkup and Mulholland [18]. This data is shown in Figure 9. The results of the regression analysis for seven calibration equations are listed in Table 6. Figure 10 shows the residual plots for the calibration equations. Only the ERTM equation with intercept and the power equation with interceptgivese a uniform distribution for the residual plots. The power equation with intercept gives smaller values for *s* and *PRESS*. This equation is adequate for this calibration curve.

The respective values for the fitting criterion, *s*, for the quadratic polynomial equation, the ERTM equation with intercept and the power equation with intercept with intercept are 0.187, 0.186 and 0.165. The respective values for the prediction criterion, *PRESS*, for these three equations are 0.599, 0.567 and 0.349. Compared with the linear equation and other polynomial equations, the results show that the power equation with intercept gives a significantly better fit and prediction.

The study by Kirkup and Mulholland [18] used three calibration equations: *y* = *a* + *bx*, *y* = *a* + *bx* + *cx*^2^ and *y* = *a* + *bx^c^*, to evaluate the fit of the calibration equations. The criteria for comparison are *R*^2^, *R_adj_*^2^ and *AIC*. Their results of the study show that there is no significant difference in the three criteria for the three calibration equations so the other two equations only give a slightly better fit than that the linear equation. However, our study uses *s* and *PRESS* as the criteria for fitting and prediction and the residual distributions were observed. 

The power equation with intercept gives a significantly better fit and prediction and is the best equation. Different criteria make the different results. 

#### 3.2.5. Evaluation of Other Data Sets

**Figure 11:**
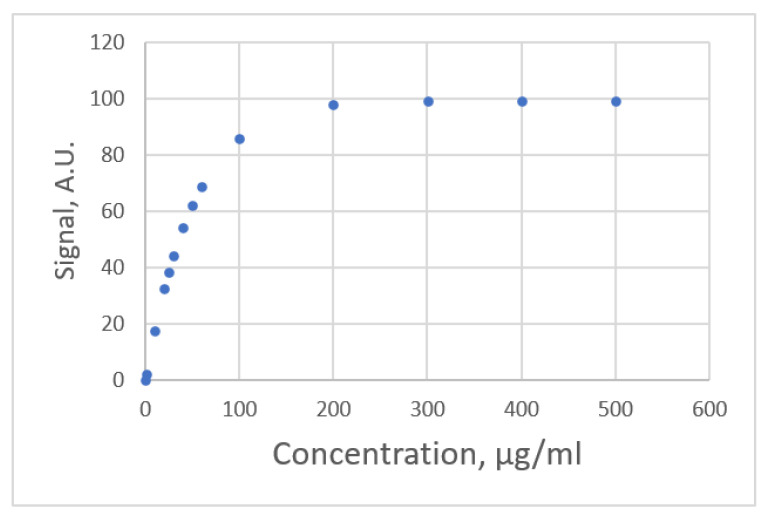
The relationship between the signal and concentrations detected by an immunoassay (data published [26], Ex.1).

Other studies show that nonlinear equations give a good fit and prediction performance. The data distribution for a study of the signal (*μA*) that is detected by an immunoassay ([26], Ex.1) is shown in Figure 11.

This distribution has the form of the ETRM curve. The response increases as the concentration increases and achieves a maximum value. The best calibration equation is:

*y* = 99.379(1 − *Exp*(−0.0197*x*)), *s* = 0.181, *PRESS* = 0.611 (20)

Martin et al. established the VitninB_12_ calibration curves and proposed the calibration equation: $\sqrt{y}=g_{0}+g_{1}logx$ [24]. However, this equation cannot be used for the blank test (*x* = 0). We evaluates the data sets using regression analysis with Equations (1)–(6). The best calibration equation is:

*y* = −0.586 + 1.173*x*^0.319^, *s* = 0.0481, *PRESS* = 0.116 (21)

The calibration curve for the Diadem concentration and the peak area for HPLC analysis was plotted by Mulholland and Hibbert [16]. The simple equation, *y* = *x*^1.1^ is used and the *R*^2^ value is 0.999. However, the residual plots exhibit a fixed pattern. The data sets are evaluated using regression analysis with Equations (1)–(6) and the best equation is:

*y* = −0.417 + 182.167(1 − *Exp*(−0.0169*x*)), *s* = 0.225, *PRESS* = 2.159 (22)

The best equation is evaluated using different regression equations, and the results are different from those of Mulholland and Hibbert [16].

### 3.3. Calibration Equations with Non-Constant Variance

#### 3.3.1. The Data Set of Lavagnimi and Magno 

**Figure 12:**
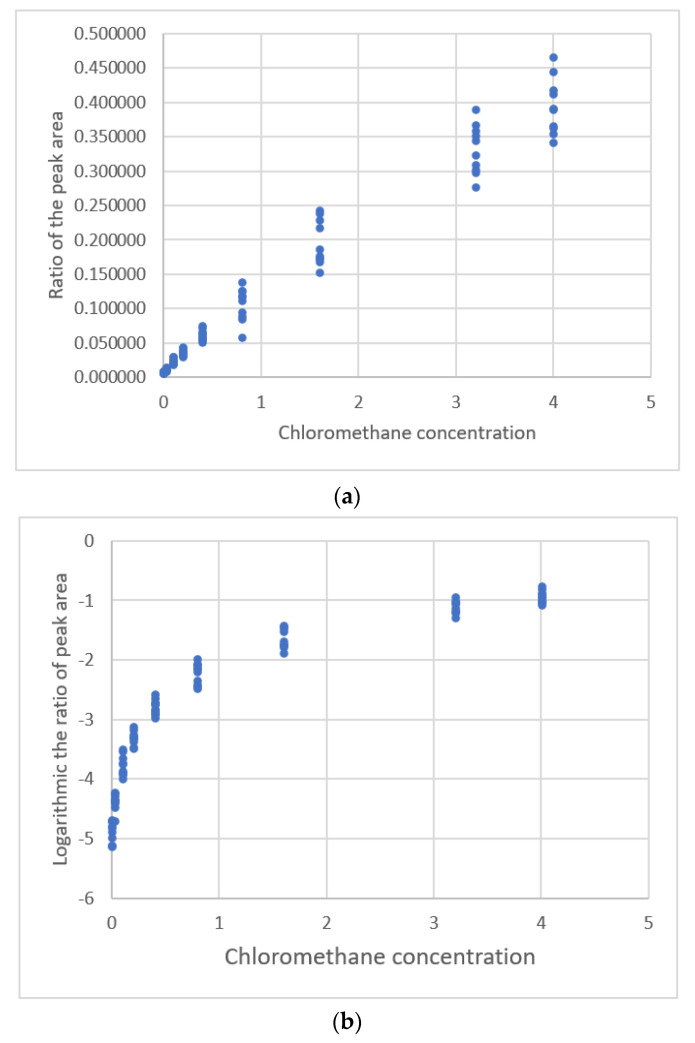
The relationship between the ratios of the peak area for chloromethane and the internal fluorobenzene standard detected using GC-MC and chloromethane concentration (data published [20]). (**a**) The ratios of peak area. (**b**) Logarithmic the ratio of peak area.

**Figure 13:**
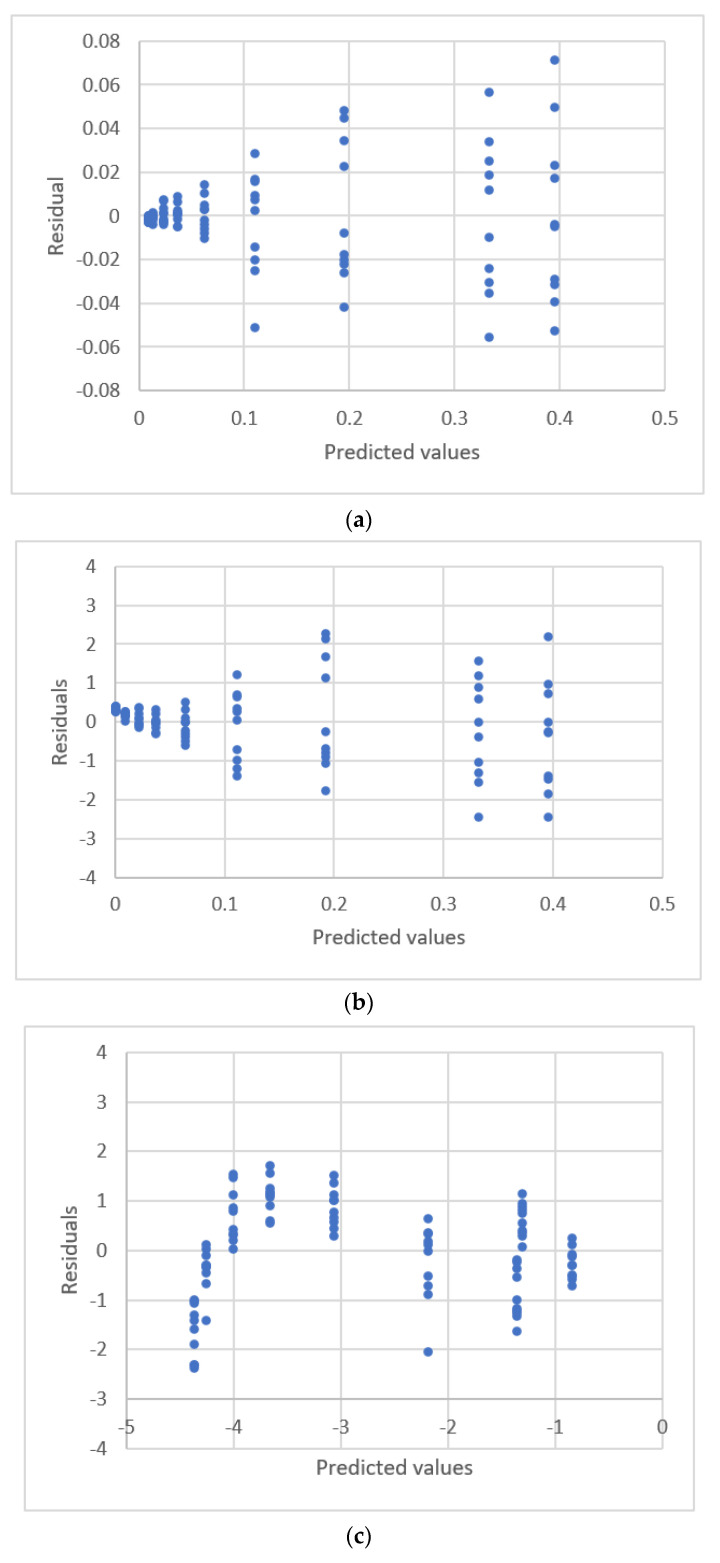

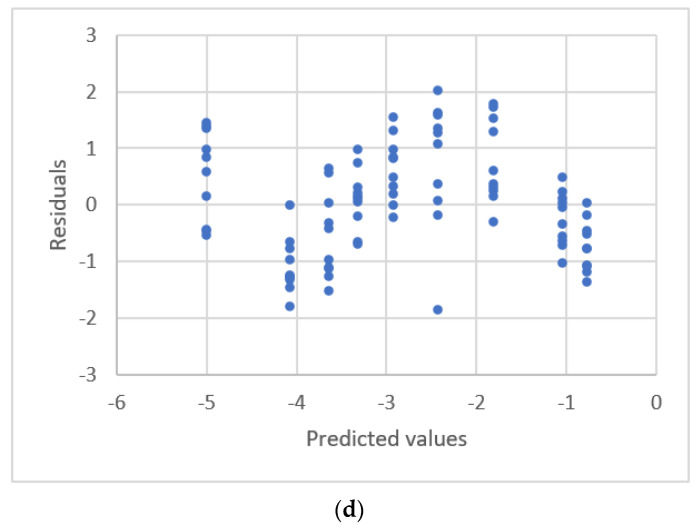
Residual plot for chloromethane concentration and the peak area of the ratios of peak area for chloromethane and the internal fluorobenzene standard using calibration equations for the data [20]. (**a**) The third polynomial equation using original *y*-values. (**b**) The power equation using original *y*-values. (**c**) The third polynomial equation using logarithmic *y*-values. (**d**) The power equation using logarithmic *y*-values.

The study by Lavagnimi and Magno [20] measured the ratio of the peak area for chloromethane and the related standard results for GC-MC. The distribution for this calibration data is shown in Figure 12a. The data becomes more scattered at the same standard level as the standard chloromethane concentration increases. The residual plots for the regression results for the calibration equations are shown in Figure 13a,b. These show a funnel pattern, so all equations give a heterogeneous variance. The results of the regression analysis for the *y*-values and standard concentration are listed in Table 7.

The replicates of the measurement of the fixed concentration levels are <9, so the weights for the weighted regression analysis cannot be calculated [33,44]. The logarithmic transformation of the dependent variable (*y*), *lny* is the new variable. The distribution between *lny* and the standard concentration is shown in Figure 12b. The data distribution converges, so the transformation stabilizes variance in the data.

The results of the regression analysis for the *lny* values and standard concentration are listed in Table 8.

Three calibration equations give a uniform distribution for the residual plots. The residual plots for two equations are shown in Figure 13c,d. The power equation with intercept gives the smallest values for *s* and *PRESS*, so this equation is the best equation for this calibration curve.

The adequate calibration equation is:

*lny* = −5.013 + 2.763*x*^0.309^ (23)

The *lny* is transformed back to the natural unit.

*y* = *Exp*(−5.013 + 2.763*x*^0.309^) *y* = 6.654 ∗ 10^−3^
*Exp*(2.766*x*^0.310^) (24)

In Table 7, the criteria, *s* and *PRESS*, are calculated using the original *y*-value but three criteria are calculated using the *lny* values in Table 8. It is inappropriate to compare the results in Table 7 and Table 8 because *y* and *lny* are dependent variables.

#### 3.3.2. Other Cases Using the Transformation of the *y*-Value

**Figure 14:**
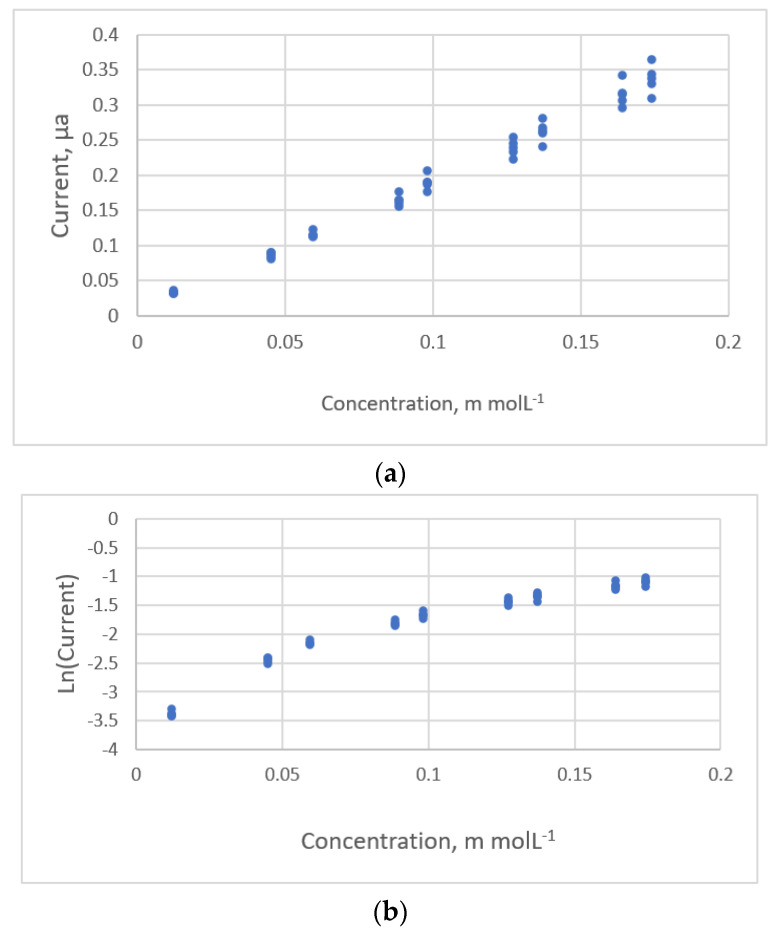
The relationship between the concentration of the benzaldehyde and the current detected for pulse polarography (data published [21]). (**a**) Current. (**b**) Logarithmic current.

The other cases that involve the transformation of *y*-value to stabilize the variance are shown in Figure 14a. The current response for pulse polarography for different concentrations of the benzaldehyde was measured by Ortiz et al. ([21], Ex.4) using a weighted linear equation, but there were only four replicates for each concentration so it is not appropriate to calculate these weights using the standard deviation.

**Figure 15:**
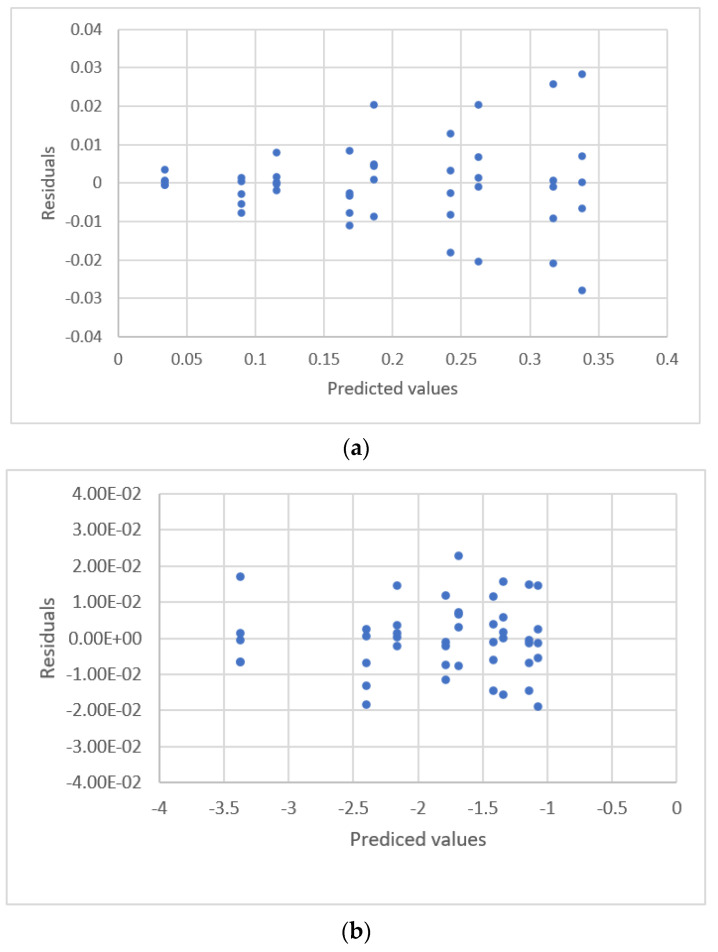
Residual plot for the concentration of the benzaldehyde and the current using calibration equations for the data [21]. (**a**) The second polynomial equation using original *y*-values. (**b**) The power equation with intercept using logarithmic *y*-values.

Six calibration equations are evaluated for these data sets. The typical residual plots for the original *y*-value are shown in Figure 15a. The funnel pattern indicates a non-constant variance in the *y* response.

When the current data is transformed, the data distribution between *lny* and the concentration is shown in Figure 14b. The typical residual plots are shown in Figure 15b.

The acceptable calibration models are:

*lny* = −3.807 + 39.695*x* − 237.956*x*^2^ + 575.645*x*^3^, *s* = 0.0506, *PRESS* = 0.130 (25)

*lny* = −6.270 + 7.605*x*^0.218^, *s* = 0.0498, *PRESS* = 0.120 (26)

The equation for natural unit *y* is:

*y* = 0.00189*Exp*(7.605*x*^0.218^) (27)

In some cases, the numeric value of response *y* is negative. These numerical values cannot be treated with the logarithmic transformation.

In a study of anti-Ig6 detection using Biophotonic sensing cells ([26], Ex.2), the transduction signal has zero values. All *y*-values are modified as *y’* = *y* + 10, and an adequate calibration equation was established:

*lny’* = −1.863 + 3.489(1 − *Exp*(−0.0943*x*)), *s* = 0.239, *PRESS* = 4.037 (28)

Transformed back to the original units, the new equation is:

*y* = −10 + 0.155 *Exp*(3.489(1 − *Exp*(−0.0643*x*)) (29)

Yang et al. established the calibration curve for the detection of cd(114) using ICP-MP and used a quadratic polynomial calibration equation [46], but the residual plots exhibit a funnel pattern. The original data sets include some data for responses with a minus sign between −7 to −53.9.

The adequate equation was evaluated as:

*lny’* = *ln*(*y* + 60) = 1.939 + 1.115(1 − *Exp*(−0.203*x*)), *s* = 0.417, *PRESS* = 15.35 (30)

The natural unit for this calibration equation is:

*y* = −60 + 6.955*Exp*(1.115(1 − *Exp*(−0.203*x*)) (31)

Bruggemann et al. plotted a calibration curve for the detection of the arsenic content using an ICP spectrometer and used a second-order polynomial equation with the criteria of *R*^2^ and the *s* value [19].

However, the residual plots exhibit a funnel pattern. The response value has a negative value from −31 to −92.

The adequate equation is:

*lny’* = *ln*(*y* + 100) = 3.512 + 4.412*x*^0.185^, *s* = 0.42, *PRESS* = 5.28, *s* = 0.42, *PRESS* = 5.28 (32)

The natural unit for this calibration equation is:

*y* = −100 + 33.529*Exp*(4.412*x*^0.185^) (33)

### 3.4. Calibration Curves with Outliers

**Figure 16:**
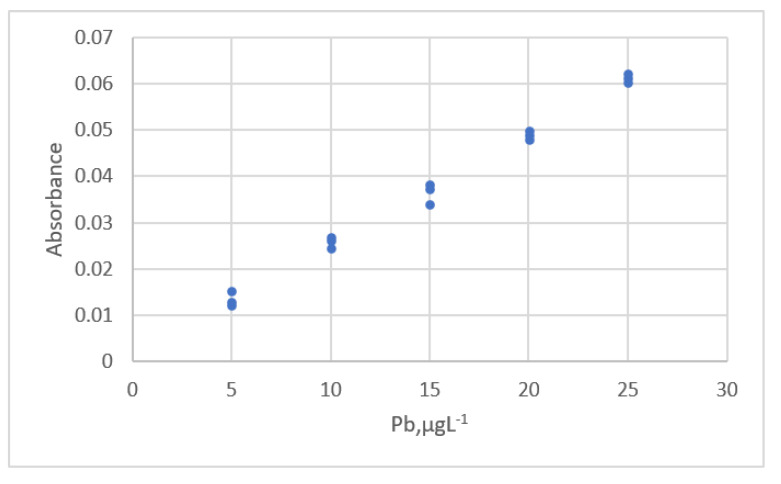
The relationship between Pb concentration and the absorbance detected by atomic absorption spectrometry (data published [41]).

Njaka et al. measured lead concentration using graphite furnace atomic absorption spectrometry [41]. The distribution between the response for absorption and the standard concentration is shown in Figure 16. It is not easy to determine a suspected outlier visually so outliers were identified using an *F*-test [41].

The results for the linear calibration equation are:

(34) $$y=0.00152+0.00237x,\;s=0.00137,\;\mathrm{PRESS}=3.136\times 10^{-5}$$

**Figure 17:**
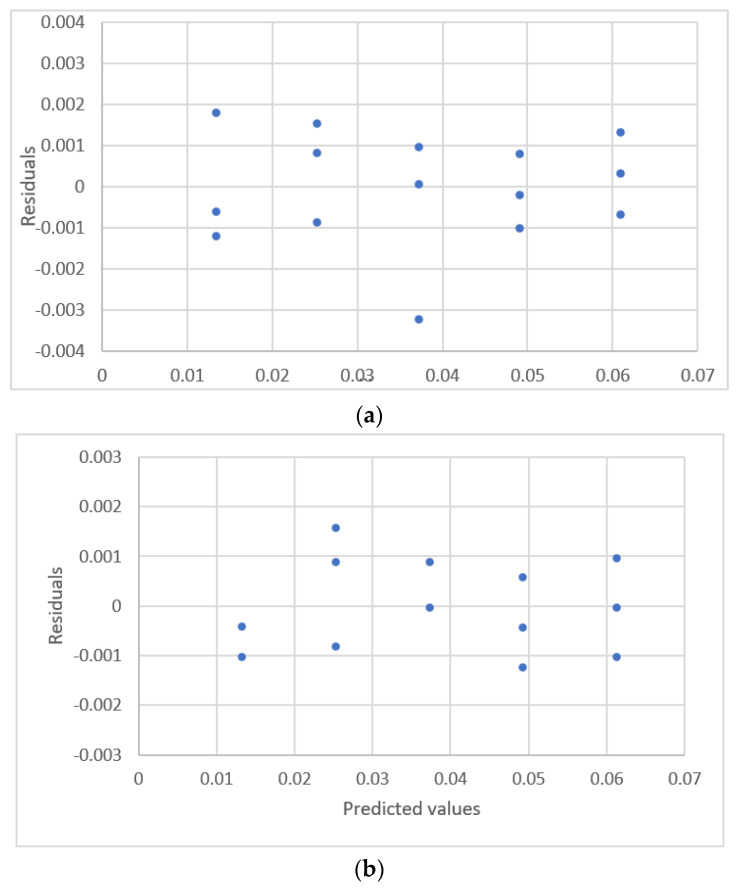
Residual plot for Pb concentration and the absorbance detected by atomic absorption spectrometry using calibration equations for the data [41]. (**a**) Linear equation with outlier. (**b**) Linear equation without outlier.

The residual plot for Equation (28) is shown in Figure 17. An outlier is reconfirmed. For this study, the statistics for this observation are verified using the standardized residual value and the *DFFITS* value. The results show that the observation is an outlier.

If the outlier is removed from the data sets, the new calibration equation is:

(35) $$y=0.00121+0.00240x,\;s=0.0095,\;\mathrm{PRESS}=1.868\times 10^{-5}$$

If the outlier is removed, the intercept and slope values are changed. The fit criterion increases from 0.00137 to 0.0075, and the prediction criterion decreases from 3.136 × 10^−5^ to 1.869 × 10^5^, so removing the outliers significantly increases the accuracy of the fit and prediction. 

**Figure 18:**
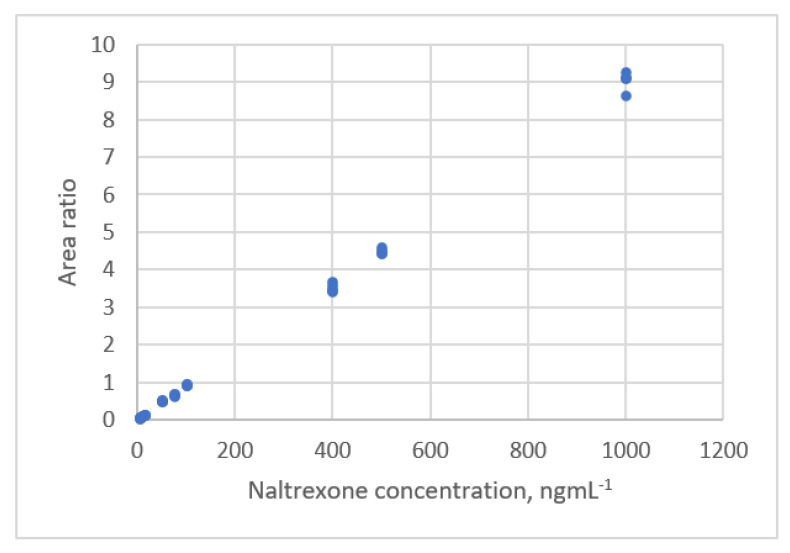
The relationship between Naltrexone concentration and the area ratio detected by LC-MS-MS (data published [17]).

The calibration curve for the area ratio for LC-MS-MS and the standard Naltrexone concentration was plotted by Desharnais et al. [17]. The measurement data is shown in Figure 18. A partial *F*-test was used to select the order of the polynomial equation, and a linear calibration equation was evaluated to be the best equation.

**Figure 19:**
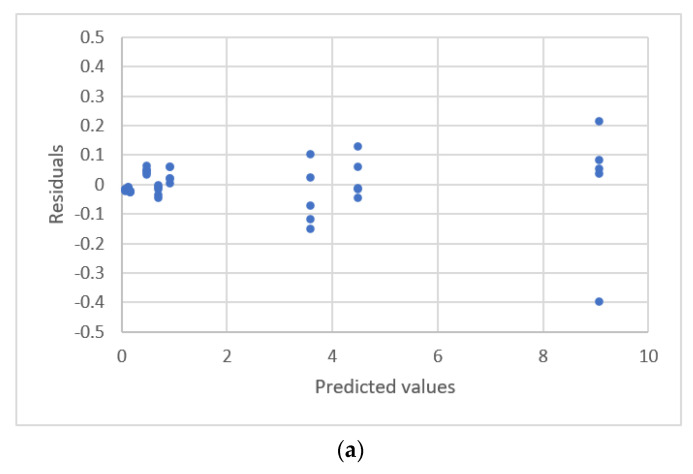

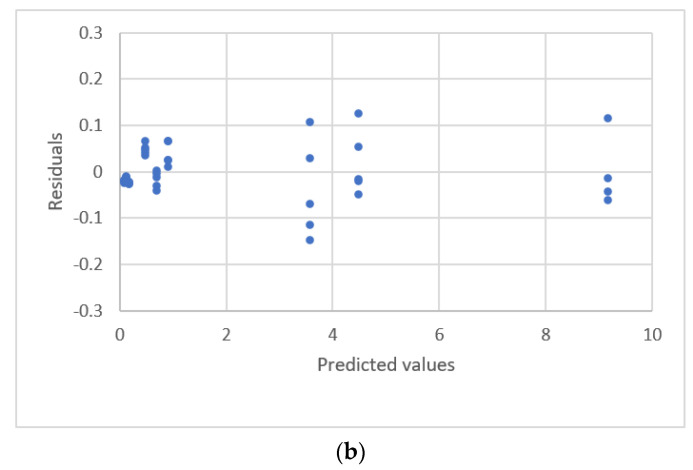
Residual plot for Naltrexone concentration and the area ratio detected by the LC-MS-MS using calibration equations for the data [17]. (**a**) The second polynomial equation with outlier. (**b**) The second polynomial equation without outlier.

The data sets were analyzed using a regression technique. A quadratic polynomic equation is the best equation and an outlier is identified. The residual plots are shown in Figure 19a.

The second polynomial equation for all data is:

(36) $$y=0.0240+0.00875x+2.806\times 10^{-7}x^{2},\;s=0.087,\;\mathrm{PRESS}=0.459$$

When the outlier was removed from the data sets, a new calibration equation was established:

(37) $$y=0.0270+0.00866x+4.639\times 10^{-7}x^{2},\;s=0.055,\;\mathrm{PRESS}=0.156$$

The residual plots for Equation (37) is shown in Figure 19b. The residuals have a uniform distribution. The fit and prediction are significantly improved using Equation (37).

**Figure 20:**
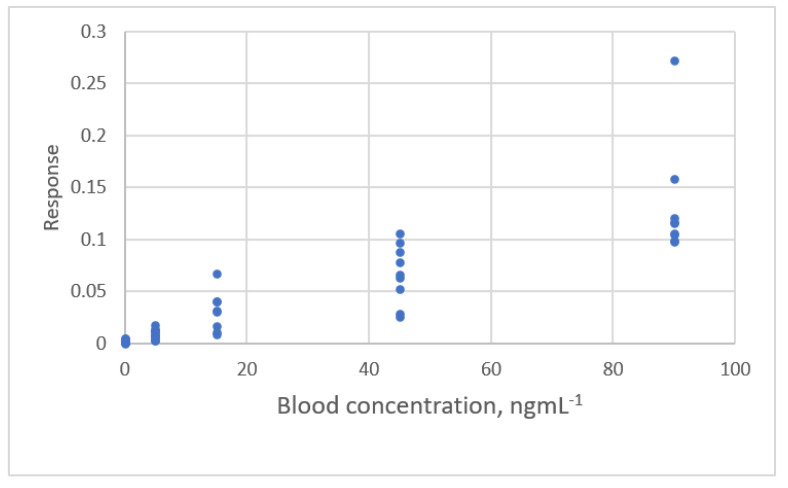
The relationship between blood concentration and the response detected by a HPLC array with a suspected outlier (data published [24]).

The value of *s* decreases from 0.087 to 0.055 and *PRESS* decreases from 0.459 to 0.156. A study by Martin et al. ([24], Ex.2) measured blood concentration using HPLC. The calibration curve is shown in Figure 20. An observation (90, 0.0272) was found.

**Figure 21:**
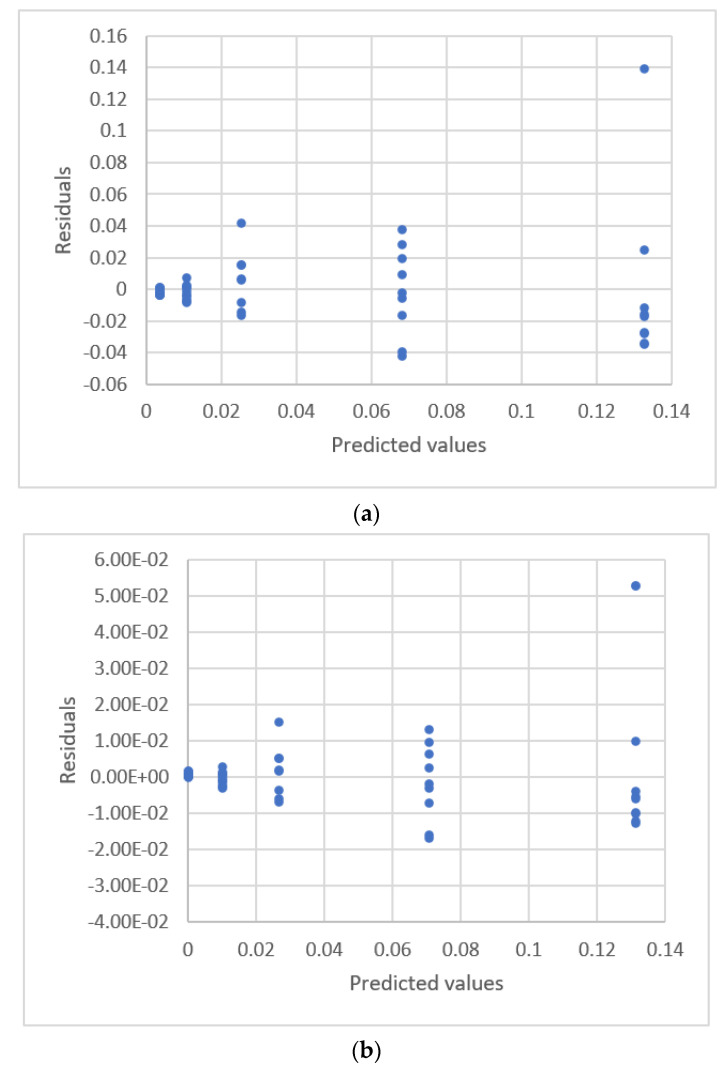
Residual plot for blood concentration and the response detected by a HPLC array for the data of Martin et al. with a suspected outlier [24]. (**a**) Linear equation. (**b**) Power equation.

The results for the fit for the calibration equations and the criteria for these calibration curves are listed in Table 9. Linear, ERTM and power equations are adequate equations, but the results of the outlier test show that the observation (90, 0.272) is an outlier. The residual plots for two equations are shown in Figure 21.

**Figure 22:**
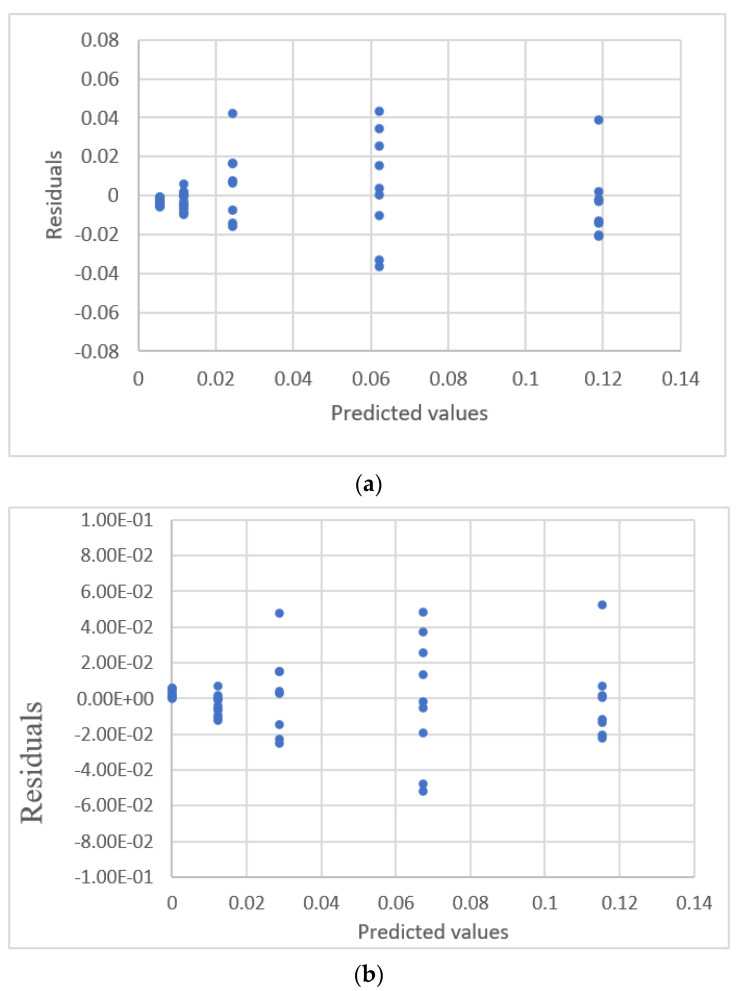
Residual plot for blood concentration and the response detected by a HPLC array for the data [24] for which the outlier is removed. (**a**) Linear equation. (**b**) Power equation.

When the outliers are removed, the calibration equation is established and the results are listed in Table 10. The residual plots are shown in Figure 22.

A comparison of Table 9 and Table 10 shows that deleting outliers improves the fit and prediction performance significantly. A calibration curve and adequate calibration equations are necessary for chemical analysis. The effect of outliers on the calibration Equation was only measured by the study of Njaka et al. [41]. For this study, outliers in three calibration curves are used to show the effect of these outliers on the fit and prediction performance and the parameter values for the calibration equations. A regression analysis technique improves the calibration equations for chemical analysis.

### 3.5. The Adequate Calibration Equations with the Data Sets Obtained with Same Equipment and Laboratory

#### 3.5.1. Data Sets of Desharnais et al

Desharnais et al. prepared the standard materials of cocaine and naltrexone in bovine blood at concentrations ranged from 5 to 1000 ng/ml. These samples were analyzed on an HPLC equipment with q mass spectrometer. There were five replicates at each concentration and the dependent variable (*y*) was the response area ratio. The adequate calibration equations for cocaine and naltrexone were:

1. Cocaine
  *y* = 29.979(1 − *Exp*(−0.0008*x*)) (38)
2. Naltrexone
  (39) $$y=0.0270+0.00866x+4.639\times 10^{-7}x^{2}$$

The adequate calibration equation was the exponential rise to maximum equation for cocaine and second-order polynomial equation for naltrexone. Both data sets were detected from the same equipment and laboratory. However, the form of the adequate calibration equations were difference.

#### 3.5.2. Data Sets of Kirkup and Mulholland

Five standard solutions were prepared and measure with HPLC by Kirkup and Mulholland [18]. The adequate calibration equations for each standard solution were listed as follows:

1. Ibuprofen
  *y* = 0.640 + 3.935*x*^0.953^ (40)
2. Genisten
  *y* = −0.640 + 3.935*x*^0.953^ (41)
3. Biovhanin
  *y* = −0.475 + 835.467(1 − *Exp*(−0.0042*x*)) (42)
4. Pseudoephedrine
  *y* = 1930.801 + 430.374*x* + 0.153*x*^2^ (43)
5. Sodium nitrate
  *y* = 8263.744*x*^1.033^ (44)

The adequate calibration equation was the power equations with intercept for Ibu profen, Genisten and Sodium nitrate, the exponential rise to maximum equation with intercept for Biovhanin and the second-order polynomial equation for Pseudoephedrine. These data sets were measured by using the same equipment in a laboratory. No universe calibration equation could be found.

#### 3.5.3. Data Sets of Martin et al

Eight standard concentrations of the compounds were prepared by Martin et al. [31]. These standard reagents were detected by using an HPLC system. There were four replicates at each concentration and the dependent variable (y) was the response area ratio. The adequate calibration equations for these reagents were listed as follows:

1. Mep
  *y* = 207336558.2*x*^0.00000472^ (45)
2. HBCDD
  *y* = 105.9236*x*^1.0132^ (46)
3. PFOS
  *y* = 32006.765 + 2267.574*x* − 6.689*x*^2^ (47)
4. PFPeA
  *y* = 20650.147 + 8660.301*x* − 4.0249*x*^2^ − 0.0011*x*^3^ (48)
5. PrP
  *y* = −18410.374 + 10679965.5 (1 − *Exp*(−0.0002*x*)) (49)
6. PFHpA
  *y* = 187092.785 + 26629692.6 (1 − *Exp*(−0.0008*x*)) (50)
7. EtP
  *y* = 2768.698 + 1231.0322*x* − 0.493*x*^2^ − 0.0030*x*^3^ (51)
8. PFOA
  *y* = 46279.18 *x*^0.773^ (52)

The adequate calibration equations included the power equation, the higher order polynomial equation and the exponential rice to maximum equation with intercept. No universe calibration equation could be found for these data sets.

## 4. Discussion

An adequate calibration equation is necessary to determine calibration curves for chemical analysis. Seventeen data sets from previous studies are used to evaluate calibration equations. A linear calibration equation can be used for two data sets only.

Nonlinear equations are suited to most of the data sets. The data distribution becomes more diverse as the standard concentration increases so a logarithmic transformation of the response is used to stabilize non-constant variance in the response data.

Linear equations are the most commonly used equations and high- order polynomial equations are used for nonlinear calibration curves. *R*^2^ is the sole criterion and the numerical value is usually very high.

This study uses a regression analysis technique. The criteria to assess the fitting-agreement are the value *s*. The predictive ability of these equations is measured in terms of the *PRESS* value. The residual plots are used as quantitative criterion to assess the adequacy of the calibration equations. As the fit and prediction ability are the principal requirement for adequate calibration equation, linear and higher order polynomial equations are not suited to many data sets.

A suspected outlier in the data sets is verified using the standardized residual and the difference in fit in standard (*DFFITS*). When the outlier is removed, the fit and prediction ability of the calibration equation improve significantly.

If an outlier is found in the standardized residual or *DFFITS*, deleting these observations improves the fit and prediction ability. However, a dominant data point provides an insight into this calibration procedure. These problems may be due to sample preparation, instrumentation adjustment or an operator’s mistake. Outliers is are identified to remove suspected observation points to improve the calibration equation and to highlight problems with the calibration procedure.

In a study of calibration equations for several compounds of environmental concern that are detected by LC-MS/MS, Martin et al. used linear and higher order polynomial equations to verify the adequacy of equations and concluded that there is no perfect model for all calibration curves [24]. This study uses four forms of calibration equations.

There is no universal equation for all calibration curves. Each calibration curve uses specific calibration equation.

In a study of the uncertainty of humidity sensors, Lu and Chen [47] found that an adequate calibration equation decreases the measurement uncertainty significantly. An evaluation of the measurement uncertainty shows that nonlinearity is the main effect on measurement uncertainty and this is mitigated by using an adequate nonlinear calibration equation [48,49].

Regression analysis is used to establish a liver volume prediction equation [50], in order to evaluate the environmental factors that affect plant tissue culture [51], to describe the water activity equations for honey [52] and to express the factors that affect the dielectric properties of foods [53]. This statistical technique is also used to evaluate adequate calibration equations for the calibration curves in this study.

A calibration curve is necessary for chemical analysis. The method that is proposed by this study can be used for other chemical instruments to establish adequate calibration equation and improve performance.

## 5. Conclusions

This study uses seventeen data sets from previous studies to evaluate the adequacy of calibration equations. Four types of calibration equations were proposed and the standard error of the estimate errors, *s* is use as the criteria to evaluate the fitting performance. The prediction ability is determined using the Prediction Sum of Squares, *PRESS* statistic. Constant variance test was performed to assess the suitable of calibration equation for this dataset. Suspected outliers in the data sets are verified.

The results of this study show that linear and higher order polynomial equations are only suitable for some data sets. Nonlinear equations, exponential rise to maximum and power equations are adequate calibration equations for others data sets. A logarithmic transformation of the response is used to stabilize non-constant variance in response data. Removing outliers significantly improves the fit and prediction ability of calibration equation. The adequate calibration equations with the data sets obtained by using same equipment in a laboratory indicated that the form of the adequate calibration equations were difference. There is no universal calibration equation for different calibration curves. The regression technique that is used in this study can be applied to other chemical instruments to establish adequate calibration equations.

## Figures and Tables

**Table 1:** Published data for evaluating the adequate calibration equations in the literature.

Study	Equipment	Target	Standard, Range	Response Range	Calibration Equation	Statistic Criteria
Mulholland and Hibbert [16]	HPLC ^1^	Diadzen	0.162–10.96 mg/50 mL	0.243–30.75 Peak area	Linear y = X^1.1^	R^2^, Residual plot
Desimoni [17]	Flow injection analysis	sulfides	0.88–81.2 μm	0.170–15.94 μA	linear	R^2^, Residual plot
Yang et al. [46]	ICP-MP ^2^	CD(114)	0–25,000 ng/L	−53.9–25,726	polynomial	Outliers, s
Bruggemann et al. [19]	ICP Spectrometer	Aresenic	0~10.0 ng/L	−92~26,394	Linear polynom	R^2^, s, Lack of fit, Residual plot
Lavagnini & Magno [20]	GC MS ^3^	Chloromethanre	0~4 μg/L	0.111975~ 0.465813 Peak area ratio	Linear polynomial	s, Residual plot
Ortiz et al. [21]	Ex1. HPLC-DAP ^4^ Ex2. Anodic Stripping voltammetry	Ascorbic Cadmium	0.004–0.026 mg/L 20.18~60.08 nmol/L	14.54–83.5 Peak area 4.50~15.98 nA	linear linear	s ANOVA, R^2^,Residual plots
	Ex3. SWADS using DMG ^5^	Nickel	0~415 μmol/L	2.5~76.87 μA	linear	R^2^
	Ex4. Pulse Polarography	Benzaldehyde	0.0198~0.1740 mnol/L	0.033~0.366 μA	linear	Residual plots, s
Kirkup and Mulholl-and [18]	HPLC	Ibuprofen Genisten Biochanin Pseudoephedrine Sodium nitrate	103.9~305.7 ng 0.159~10.16 mg 0.158~10.09 mg 61.4~181.5 mg 1.006~25.16	Area 261.357~755.89 0.15508~35.2175 0.12111~34.0687 28,653~85,241 8103~233,405	Linear polynomicl Y = a + bx^m^	R^2^, Radj^2^, ANOVA, AIC ^9^, residual plots
Rawski et al. [22]	Spectrophotomethic	Albumin	0~20 μg/mL	0~450 Peak height × 10^−3^	Linear	Lack of fit, R^2^
Desharnais et al. [17]	LC-MS ^6^	Cocaine Naltrexone	5~1000 ng/mL 5~1000 ng/mL	0.049~9.209 Area ratio 0.226~16.298 Area ratio	Linear linear	Partial *F*-test, ANOVA Partial *F*-test, ANOVA
Martin et al. [24]	Ex1. HPLC	Vitamin B_12_	0.23~4.0 ng	0.14~1.29 Area ratio	High order polynomial	R^2^ Residual Plots
	Ex2. HPLC	Blood	0~90 ng/mL	0.002~0.272 Area ratio		
Martin et al. [31]	LC-QqQ-MS ^7^	MeP	1–1500 ng/mL	864–1,470,121	linear	R^2^
	arsay	HBCDD	1–1500 ng/mL	105–175,247		
		PFOS		2548–1,924,470		
		PFPeA		9110–7,597,353		
		PrP		2150–3,054,469		
		PFHpA		29,847–19,417,533		
		EtP		1007–2,062,210		
		PFOA		12,569–12,906,640		
Njaka et al. [41]	GF-AAS ^8^	Pb	5~25 μg/L	0.0122~0.0622 Absorbanc	linear	R^2^ Outliers., Residual plots.
Lavin et al. [26]	Ex1.Jmmunoassay moden	unknow	0~500 μg/mL	0~99.2	polynomial	AICs ^10^, R^2^
	Ex2. Biophotonic sensing cells	Anti-IgG	1~100 μg/mL	0.00~6.14	polynomial	AICs, R^2^

Note: ^1^. HPLC: High-performance liquid chromatography. ^2^. ICP-MP: Inductively Coupled Plasma Mass Spectrometry. ^3^. GC–MS: Gas chromatography/mass spectrometry. ^4^. HPLC-DAP: High performance liquid chromatography-diode array detection. ^5^. SWADSV using DMG: Square-ware adsorptive-Stripping voltammetry using dimethyl lyoxine. ^6^. LC-MS: Liquid chromatography-tandem mass spectrometry. ^7^. LC-QqQ-MS: liquid chromatography–mass spectrometer. ^8^. GF-AAS: Graphite furnace atomic adsorption spectrometry. ^9^. AIC: Akaikes information criterion. ^10^. AICs: The small sample adjusted information criteria.

**Table 2:** The evaluation of fitting calibration equations and criteria for the ascorbic concentration and the peak area for HPLC (data published [21]).

Equation	s	PRESS	Residual Plots
1. y = 222.512 + 30812x	89.644	160,637	Uniform distribution
2. y = 191.243 + 313946.6x − 199117.011x^2^	92.034	182,248	U.D.
3. y = 48234.378(1 − Exp(−7.150x))	102.857	220,533	U.D.
4. y = 188.052 + 222889.55(1 − Exp(−1.4113x))	92.037	182,214	U.D.
5. y = 255791.66x^0.9426^	92.241	176,843	U.D.
6. y = 160.955 + 290978.8x^0.983^	92.017	179,136	U.D.

Note: U.D.: Uniform distribution.

**Table 3:** The evaluation of fitting calibration equations and criteria for sulfide concentration and the response for flow injection analysis (data published [17]).

Equation	s	PRESS	Residual Plots
1. y = −0.172 + 2.818x	0.315	4.140	Fixed pattern
2. y = −0.414 + 3.0801x−0.0241x^2^	0.225	2.418	U.D.
3. y = 624.382(1 − Exp(−0.0046x))	0.335	4.498	Fixed pattern
4. y = −0.417 + 182.166(1 − Exp(−0.0169x))	0.225	2.259	U.D.
5. y = 2.806x^0.998^	0.334	4.814	Fixed pattern
6. y = −0.543 + 3.237x^0.945^	0.241	2.472	U.D.

**Table 4:** The evaluation of fitting calibration equations and criteria for nickel concentration and the current for square-wave adsorptive-stripping voltammetry (data published [21]).

Equation	s	PRESS	Residual Plots
1. y = 9.832 + 192.469x	7.587	1299.3	Fixed pattern
2. y = −2.861 + 383.458x − 459.414x^2^	2.986	231.49	Fixed pattern
3. y = 1.620 + 235.042x + 452.872x^2^ − 1455.64x^3^	1.707	81.364	Fixed pattern
4. y = 3.347 + 124.632x + 1733.643x^2^ − 6367.25x^3^ + 5939.812x^4^	1.360	70.125	U.D.
5. y = 103.107(1 − Exp(−3.712x))	3.982	361.11	Fixed pattern
6. y = −2.975 + 101.749(1 − Exp(−4.150x))	3.966	434.6	Fixed pattern
7. y = 155.549x^0.686^	5.692	740.4	Fixed pattern
8. y = −3.401 + 155.388x^0.644^	5.716	1919.1	Fixed pattern

**Table 5:** The evaluation of fitting calibration equations and criteria for Albumin concentration and the response for spectrophotometric measurement (data published [22]).

Equation	s	PRESS	Residual Plots
1. y = 47.773 + 22.047x	27.408	7431	Fixed pattern
2. y = 4.946 + 36.322x − 0.714x^2^	8.766	2718	U.D
3. y = 617.147(1 − Exp(−0.0654x))	8.560	2611	U.D
4. y = 0.707 + 618.310(1 − Exp(−0.650x))	8.698	2672	U.D
5. y = 57.694x^0.696^	12.781	5976	Fixed pattern
6. y = −7.232 + 61.790x^0.678^	12.766	5948	Fixed pattern

**Table 6:** The evaluation of fitting calibration equations and criteria for ibuprofen concentration and the peak area for HPLC (data published [18]).

Equation	s	PRESS	Residual Plots
1. y = −0.670 + 3.510x	0.280	1.253	Fixed pattern
2. y = −0.473 + 3.731x − 0.022x^2^	0.187	0.599	Fixed pattern
3. y = −6319 + 4.074x − 0.125x^2^ + 0.007x^3^	0.148	0.348	Fixed pattern
4. y = 2128.294(1 − Exp(−0.0016x))	0.356	1.595	Fixed pattern
5.y = −0.477 + 296.738(1 − Exp(−0.0126x))	0.186	0.567	U.D.
6. y = 3.443x^1.004^	0.354	1.737	U.D.
7. y = −0.640 + 3.935x^0.953^	0.165	0.349	U.D.

**Table 7:** The evaluation of fitting calibration equations and criteria for the ratio of peak area for chloromethane and of the internal fluorobenzene standard detected by GC-MC and chloromethane concentration using the y variable (data published [20]).

Equation	s	PRESS	Residual Plots
1. y = 0.0186 + 0.0972x	0.0243	0.0554	Fixed pattern
2. y = 0.010133 + 0.128x − 0.00804x^2^	0.0320	0.0475	Fixed pattern
3. y = 0.664 (1 − Exp(−0.222x))	0.0227	0.0501	Fixed pattern
4. y = 0.132x^0.790^	0.0221	0.0470	Fixed pattern

**Table 8:** The evaluation of fitting calibration equcations and criteria for the ratio of the peak area for chloromethane and the internal fluorobenzene standard detected by GC-MC and chloromethane concentration using the lny variable (data published [20]).

Equation	s	RESS	Residual Plots
1. Lny = −4.165 + 2.356x − 0.403x^2^	0.423	16.501	Fixed pattern
2. Lny = −4.373 + 3.837x − 1.544x^2^ + 0.201x^3^	0.320	9.494	Fixed pattern
3. Lny = −4.561 + 5.986x − 54.802x^2^ + 1.439x^3^ − 0.154x^4^	0.242	6.811	U.D.
4. Lny = −4.537 + 3.393x + 1.678x^2^	0.271	6.839	U.D.
5. Lny = −5.0126 + 2.763x^0.309^	0.224	4.578	U.D.

**Table 9:** The evaluation of fitting calibration equations and criteria for blood concentrations and the response detected by a HPLC array with suspected outlier (data published [24]).

Equation	s	PRESS	Residual Plots
1. y = 0.00364 + 0.00143x	0.0266	0.0415	U.D. with outlier
2. y = −0.504(1 − Exp(−0.00340x))	0.0267	0.0420	U.D. with outlier
3. y = 0.0024x^0.893^	0.0167	0.0411	U.D. with outlier

**Table 10:** The evaluation of fitting calibration equations and criteria for blood concentrations and the response detected by a HPLC array when outliers are deleted (data published [24]).

Equation	s	PRESS	Residual Plots
1. y = 0.00543 + 0.00126x	0.0165	0.0139	U.D.
2. y = 0.192(1 − Exp(−0.0101x))	0.0158	0.0142	U.D.
3. y = 0.00351x^0.779^	0.0161	0.0137	U.D.
